# Spatial Variation in Cd, Pb, Hg, and Zn Accumulation in Edible Wild-Growing Mushroom Species from Different Environmentally Loaded Areas in Southern Poland: Risk Assessment and Implications for Consumer Safety

**DOI:** 10.3390/toxics14010036

**Published:** 2025-12-29

**Authors:** Monika Rusin, Joanna Domagalska, Agnieszka Czendlik, Natalia Wróbel, Anna Kidoń

**Affiliations:** 1 Department of Environmental Health, Faculty of Public Health in Bytom, Medical University of Silesia in Katowice, 18 Piekarska Street, 41-902 Bytom, Poland; jdomagalska@sum.edu.pl; 2 Graduates of the Faculty of Public Health in Bytom, Medical University of Silesia in Katowice, 18 Piekarska Street, 41-902 Bytom, Poland; agatka.cze@wp.pl (A.C.); natay.wrobel@gmail.com (N.W.); s75560@365.sum.edu.pl (A.K.)

**Keywords:** heavy metals, environmental exposure, environmental pollution, food safety, health risks, public health

## Abstract

The uptake and accumulation of heavy metals by wild-grown mushrooms is raising health concerns for consumers worldwide with respect to variability conditioned by species and harvesting site specificity. This study aims to evaluate the concentration of elements (Zn) and heavy metals (Cd, Pb, Hg) in wild-growing edible mushroom samples (n = 200) collected from industrial and non-industrial areas in Poland. Over half of the analyzed mushroom samples (51%) exceeded EU limits for Cd, Pb, or Hg. Xerocomellus chrysenteron and X. subtomentosus (XCS) showed the highest accumulation, with median Cd and Pb concentrations of 3.53 mg/kg and 0.63 mg/kg fresh mass, respectively, in industrial areas. Spatial factors, including distance from emission sources and wind direction, significantly influenced element accumulation, with Cd levels in XCS up to 20 times higher than in Suillus species. A high-consumption scenario (96 g/day) indicated a substantial non-carcinogenic risk (HQ > 1) from Cd exposure via XCS consumption, both in industrial (HQ up to 9.01) and non-industrial areas (HQ max = 1.80), with cumulative hazard index (HI) ranging from 1.21 to 11.01. It is imperative to select the optimal regions for mushroom harvesting and to refrain from consuming species that accumulate elements to the greatest extent.

## 1. Introduction

In recent decades, a marked increase in the popularity of natural foods has been observed, reflecting a growing awareness among consumers of the impact of diet on health. In the context of global dietary changes, including the rejection of highly processed products and the search for minimally processed alternatives, mushrooms cultivated in their natural environment occupy a special place. In Central Europe, including Poland, forest mushrooms play a significant role in the diet of many individuals. This phenomenon is attributable not only to the preferences in taste and the recognized nutritional value of these foods, but also to the influence of culinary traditions and a passion for foraging, a practice that is particularly prevalent in rural areas [1,2]. They are regarded as a high-nutrient raw material that provides protein, fiber, vitamins, and minerals. These organisms have also become the focus of significant research interest, particularly concerning their capacity to bioaccumulate toxic elements, including heavy metals [3,4,5,6,7].

The protein content of forest mushrooms, such as *Boletus edulis* (porcini mushrooms) and *Leccinum aurantiacum* (red-capped bolete), has been found to exceed that of popular cultivated species, including *Agaricus bisporus* (button mushrooms) and *Pleurotus ostreatus* (oyster mushrooms) [2,8]. However, despite their relatively high protein content, the bioavailability of these mushrooms is limited due to the presence of chitinous components in their cell walls and the low digestibility of some of their proteins [2,9,10]. Beyond their nutritional value, mushrooms are a significant source of various bioactive compounds, which possess immunomodulatory, hypolipidemic, antibacterial, antiviral, and antioxidant properties. Of particular significance are the phenols, flavonoids, carotenoids, ascorbic acid, and indole compounds found in mushrooms, which play a pivotal role in neutralizing oxidative stress. Oxidative stress is a major contributing factor in the pathogenesis of numerous lifestyle diseases [8,9,10,11,12,13,14]. For instance, p-hydroxybenzoic acid, which has been shown to possess anti-inflammatory, antiviral, and antioxidant properties, has been detected in *Boletus edulis* and *Imleria badia* (bay bolete) [13,14,15,16]. Polysaccharides present in mushrooms, including β-glucans, which have immunostimulatory and anticancer properties, also merit particular consideration. A review of the extant literature reveals indications of the efficacy of the treatment in the management of cancers of the breast, cervical, lung, and digestive systems [12,17,18].

Macroscopic fungi possess a distinctive capacity to accumulate a diverse array of chemical elements, rendering them highly advantageous as indicator organisms in environmental studies. The extensive mycelium network characteristic of macroscopic fungi, which penetrates the litter and mineral layers of the soil, determines the effective uptake of bioelements and toxic elements present in the soil environment [4,5,19,20]. In contrast to higher organisms, mushrooms lack effective homeostatic mechanisms for numerous metals, leading to their passive uptake and subsequent accumulation within cellular structures and at the cell wall level. This phenomenon is of particular importance in relation metals with high biological toxicity, such as cadmium (Cd), lead (Pb), and mercury (Hg), as well as zinc (Zn), which, despite performing important physiological functions, exhibits toxic effects at concentrations exceeding homeostatic levels [4,5,6,19,21].

Cd, Pb, and Hg are elements that are not essential for human physiology, and their presence in the body can lead to various metabolic disorders [22,23,24]. Cd primarily accumulates in the kidneys and liver, resulting in damage to cell structures, oxidative stress, and disturbances in calcium and phosphate metabolism, including osteomalacia and kidney dysfunction. Cd has also been demonstrated to possess carcinogenic effects [22,25,26,27]. Despite substantial reductions in its emissions over the past few decades, Pb continues to be a pervasive soil contaminant, exhibiting notable neurotoxic, hematotoxic, and endocrine effects [22,28,29]. Hg, particularly in its methylated form, is characterized by its high bioavailability and neurotoxicity. It accumulates in brain tissue, resulting in permanent damage to the nervous system [22,30,31]. Zn, an essential element for the proper functioning of numerous enzymes and structural proteins, can disrupt the homeostasis of other micronutrients (particularly copper and iron) in excess. This can lead to oxidative stress and have a negative effect on metabolic functions [32,33].

Spatial variation in heavy metals and elements content in mushrooms can serve as a valuable indicator of environmental quality and the extent of anthropogenic pressure. A comparative analysis of fruiting bodies from industrial and non-industrial regions facilitates the identification of high-risk areas and the assessment of potential health risks to consumers.

The objective of this study was to evaluate spatial variation in the accumulation of selected toxic elements (Cd, Pb, Hg, and Zn) in the fruiting bodies of wild forest mushrooms collected from areas characterized by different levels of anthropogenic pressure in southern Poland. The analysis included heavily industrialized regions (Tarnowskie Góry and Olkusz counties) and non-industrialized areas (Szydłowiec and Wadowice counties), with the latter serving as reference sites for assessing environmental background levels. The objective of the study was to characterize species-specific bioaccumulation patterns and to assess the influence of selected spatial and environmental factors on toxic element concentrations in mushrooms. The selected spatial and environmental factors included distance from and position relative to sources of metal emissions. The study also aimed to identify key variables governing the accumulation of toxic elements and evaluate potential human health risks associated with consuming the studied species, in the context of food safety and soil quality assessment.

In the context of the growing demand for natural foods, regular monitoring of selected elements content in mushrooms is imperative to ensure consumer safety and reliable assessment of the quality of the natural environment.

## 2. Materials and Methods

### 2.1. Research Material

The study sample consists of 200 mushroom specimens representing 10 distinct species, collected from four counties in Poland: Szydłowiec, Wadowice, Tarnowskie Góry, and Olkusz. The sample population is distributed uniformly across two distinct categories: non-industrial (50% of the sample) and industrial (50% of the sample). The industrial category is further sub-classified based on their proximity to emission sources, with samples collected from distances of 0–200 m (49 samples) and 200–600 m (51 samples). Additionally, the geographical distribution of the industrial sites was considered, with sampling locations categorized as situated west (51 samples) or east (49 samples) of the emission sources based on their relative spatial position. Non-industrial samples were exclusively collected from the Szydłowiec and Wadowice counties (50 samples each), while industrial samples originated from the Tarnowskie Góry (50 samples) and Olkusz (50 samples). The most prevalent species in non-industrial sites was *Boletus edulis* (BE) (38%), whereas *Suillus luteus* (SL; slippery jack) and *S. grevilleai* (SG; larch bolete), and *Xerocomellus chrysenteron* and *X. subtomentosus* (XCS; red-cracking bolete and yellow-cracking bolete) predominated in industrial sites, contributing up to 65% and 62% of samples, respectively, in specific strata. The mushrooms were collected in September and October of 2024. For the purpose of analysis, only mushrooms that had not been consumed by worms were selected for study. During the process of collecting mushrooms, the following protocol was implemented to ensure the procurement of a sample that was both comprehensive and representative:

- mushroom cluster—1 sample;
- single mushrooms in close proximity (approx. 20 m)—1 sample.

The species were grouped into two categories: *Suillus luteus* and *S. grevillei* (SLG) and *Xerocomellus chrysenteron* and *X. subtomentosus* (XCS), based on their prevalence in the sampled sites and preliminary observations of similar ecological roles. The rationale for this aggregation, including statistical confirmation of comparable element accumulation profiles, is provided in Section 2.5.

#### Site-Specific Characterization of Fruiting Body Sampling Locations in Industrial and Non-Industrial Forest Areas of Southern Poland

The spatial distribution of metals pollution in the natural environment is influenced by geographical and anthropogenic factors. The local levels of toxic elements presence are shaped by geological structure, soil properties, and the intensity of industrial activity [34,35]. In regions exhibiting minimal anthropogenic influence, such as Wadowice county in Małopolska province and Szydłowiec county in Masovian province, the chemical composition of soils is distinguished by comparatively low concentrations of toxic elements, frequently falling within the geochemical background range. The forest and agricultural habitats that predominate in these regions are predominantly shaped by natural factors, with a negligible contribution from anthropogenic pollution [36,37].

The soil environment in areas subject to long-term heavy industrial influence exhibits signs of significant degradation, as evidenced in the Tarnowskie Góry county (Silesian province) and the vicinity of Bukowno (Olkusz county, Małopolska province). Tarnowskie Góry county is the location of the ‘Miasteczko Śląskie’ zinc smelter, a plant which has been in operation since 1960 and which produces Zn, Pb, and refined Cd. Olkusz county is characterized by the presence of the Bolesław mining and metallurgical plant, which has been in operation since 1955 and specializes in Zn production. Until 2020, the plant also engaged in the mining of Zn and Pb. The long-term operation of industrial facilities has been demonstrated to result in substantial contamination of metals within the living and non-living environment. This assertion is substantiated by the findings of numerous scientific studies conducted in these regions [38,39,40,41,42,43,44].

**Figure 1:**
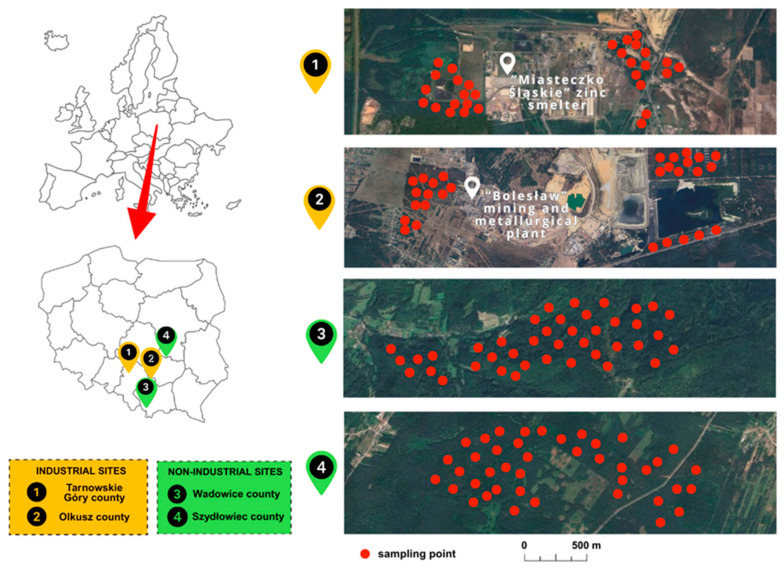
Locations of mushroom sampling sites.

The long-term extraction and processing of Zn and Pb ore has led to the accumulation of elements in the soil, primarily due to the emission of metallic dust, the deposition of flotation waste, and the infiltration of industrial wastewater. Despite a decline in industrial activity, the soils in these areas persist in their heavy contamination, with the presence of metals that exhibit high bioavailability. This poses a risk of transfer of these elements to living organisms, including mushrooms [44,45,46,47]. Figure 1 illustrates the geographical distribution of the sampling sites and the approximate location of the mushroom sampling sites. The geographical coordinates for the sampling areas are shown in Table S1—Supplementary Materials.

The number of mushroom samples of individual species, along with the locations where they were collected, is presented in Table 1.

### 2.2. Laboratory Analysis of Metals Content in Test Samples

Subsequent to the harvesting process, the mushroom samples were meticulously cleaned of surrounding litter and sand. Thereafter, they were carefully packed into separate zip-lock bags, labeled with precise identifiers, and subsequently frozen at −20 °C. The prepared research material was transported under controlled temperature conditions, maintaining an unbroken cold chain to ensure its chemical and microbiological integrity, to the Analytical Laboratory of the Department of Environmental Health at the Medical University of Silesia in Katowice for the determination of metals content for Cd, Pb, Hg, and Zn.

In the laboratory, the samples were thawed, blended, and coded. All mushroom samples were analyzed as whole fruiting bodies. The hymenium was not separated from the cap or stipe prior to analysis. This uniform sample preparation procedure was applied to all species and sampling sites to ensure methodological consistency and to reflect typical consumer practices, as edible mushrooms are commonly consumed whole. Each sample was weighed to an accuracy of 0.5 g (with a permissible deviation of ±2%) using a PS 750/X (RADWAG, Poland, Radom) precision balance. The weighed samples were then transferred to Teflon digestion vessels.

For the subsequent digestion process, 9 mL of ultra-pure nitric acid (HNO_3_; 65% ultra-pure; MERCK, Germany, Darmstadt) and 1 mL of 30% hydrogen peroxide (H_2_O_2_; 30% by Stanlab, Poland) were added to each Teflon vessel. The vessels were then sealed and placed into a multi-station microwave mineralizer (ETHOUS UP, Milestone, Italy, Sorisole), wherein the samples underwent a multistage mineralization protocol as follows:

1. Stage I: duration: 20 min., temperature: 210 °C., generator power: 1800 W;
2. Stage II: duration: 15 min., temperature: 210 °C., generator power: 1800 W;
3. Stage III: cooling process: 30 min.

At the end of the digestion process, the samples were transferred to 50 mL volumetric flasks and diluted to the required volume with ultrapure water.

The concentrations of Cd and Pb were determined by electrothermal atomization atomic absorption spectrometry (ET-AAS), a method that utilizes a Savanta Sigma spectrometer (GBC Scientific Equipment, Australia, Keysborough). This instrument is equipped with a PAL3000 automatic sample feeder and a GF3000 graphite furnace.

All collected mushroom fruiting bodies were analyzed for total Hg (Hgₜₒₜₐₗ) content, which serves as a proxy for overall Hg contamination. According to the literature, Hg in terrestrial fungi primarily occurs in inorganic form (Hg^2+^), while the proportion of methylmercury (MeHg)—the more toxic, bioavailable species—is minimal, often representing less than 5% of total Hg and frequently below analytical detection limits [48,49]. Hg content was analyzed using cold vapor (CV) technique coupled with atomic fluorescence spectrometry (AFS), using a Millennium Merlin 10.025 total Hg analyzer (PS Analytical, UK, Orpington).

The concentrations of Zn were determined by means of inductively coupled plasma atomic emission spectrometry (ICP-OES) with an Ultima Expert LT spectrometer (HORIBA Scientific, France, Palaiseau).

In order to guarantee analytical reliability, chemical analyses were performed in duplicate for each sample.

The measurement methods and conditions of the spectrometers are outlined in Table S2—Supplementary Materials.

The limit of quantification (LOQ) of the method was 0.01 mg·kg^−1^ for Cd, 0.10 mg·kg^−1^ for Pb, 0.69 mg·kg^−1^ for Zn, and 0.001 mg·kg^−1^ for Hg, while the limit of detection (LOD) was 0.005 mg/L for Cd, and 0.053 mg/L for Pb, 0.42 mg/L for Zn, and 0.0005 mg/L for Hg.

The standard solutions employed for the calibration of instruments comprised certified reference materials obtained from a range of reputable suppliers in order to ensure analytical accuracy and ability. The calibration of the spectrometers was conducted using certified AccuStandard reference solutions, with the serial numbers listed below: Cd—220065069, Hg—221065171, Pb—220055040, and Zn—220065027.

Metal concentrations are presented in mg·kg^−1^ f.m. (fresh mass/wet weight), as this allows comparison with the maximum permissible concentrations of studied elements in food set out in relevant regulations [50,51].

### 2.3. Toxicological Risk Assessment

Non-cancer risks associated with metals exposure (oral exposure) were estimated using the Average Daily Dose (ADD) formula recommended by the US Environmental Protection Agency (US EPA) [52]:

(1) $$ADD\;=\;\frac{C*IngrR*EF*ED}{BW*AT}\;[\mathrm{mg}/\mathrm{kg}/\mathrm{day}]$$

where:

ADD—Average daily potential dose of Zn/Cd/Pb/Hg ingested via the dietary route [mg/kg/day]

C—concentration of Zn/Cd/Pb/Hg in the food product [mg/g]

IngR—Ingestion rate [g/day]

EF—Exposure frequency [365 days]

ED—Exposure duration [70 years]

BW—Body weight [70 kg]

AT—Averaging time [25,550 days].

According to recent data from the Central Statistical Office in Poland, the average daily consumption of edible wild forest mushrooms per capita in Polish households is approximately 5.5 g per day [53]. However, according to information on the seasonal consumption of wild mushrooms among the population living in rural areas of large forest areas, it is 96 g per day [54]. The exposure assessment was based on two scenarios. In the first scenario, the average daily consumption of edible wild forest mushrooms per capita is 5.5 g. In Scenario 2, the average daily consumption of wild mushrooms among the population residing in rural areas of large forest areas is 96 g.

The health risk assessment for adults was performed for each element by estimating the hazard quotient (HQ) using the following formula [55]:

HQ = ADD/RfD(2)

The US EPA has established reference doses (RfD) for oral exposure, specifically 0.3 mg/kg/day for Zn [56], 0.001 mg/kg/day for Cd [57], 0.0036 mg/kg/day for Pb [58], and 0.0003 mg/kg/day for Hg [59].

To estimate the total potential non-cancer health effects due to exposure to a mixture of elements from the consumption of wild-growing mushrooms, a cumulative hazard index (HI) was calculated in accordance with the EPA Health Risk Assessment Guidelines [60]. The cumulative risk from concurrent exposure to multiple non-carcinogenic substances is determined by summing the individual HQ values to obtain the HI, which is interpreted in a manner analogous to the HQ index.

An HQ or HI value ≤1 indicates negligible risk, while values >1 suggest potential health risks [55,60].

### 2.4. Statistical Analysis

Analyses were conducted using the R statistical language (version 4.3.3; R Core Team, 2024), using the packages rio, gt, broom, gtsummary, MASS, ggplot2, dplyr, purrr, tidyr and cowplot.

Significance level was α = 0.05 for all tests. Descriptive statistics included medians and interquartile ranges (IQR) for skewed selected element concentrations (Zn, Cd, Hg, and Pb in mg·kg^−1^ f.m.), and frequencies/percentages for categorical variables (e.g., species, county, site characteristics). Shapiro–Wilk tests confirmed non-normality (*p* < 0.05 for all elements/groups). Levene’s tests indicated heteroskedasticity. Group comparisons (e.g., industrial vs. non-industrial sites, east vs. west positions, 0–200 m vs. 200–600 m distances) employed two-tailed Wilcoxon rank-sum tests (exact for *n* ≤ 5 per group). The Hodges–Lehmann estimator provided median differences with 95% CI (using bootstrap with 1000 replications). Effect size was rank-biserial correlation (r), interpreted as small (|r| < 0.3), medium (0.3–0.5), or large (>0.5). *p*-values were adjusted via the Benjamini–Hochberg false discovery rate (FDR) per table.

Relationships with environmental variables were modeled using multivariable robust linear regression, with log-transformed concentrations as outcomes. Models included main effects (distance, position, species) and interactions (distance × species, position × species), fitted separately per element. Coefficients (β) had 95% CI and *p*-values from robust standard errors; no adjustments across elements due to exploratory focus, with emphasis on effect sizes and intervals.

### 2.5. Justification for Species Aggregation

In order to address the potential variability that may be observed within grouped species, the similarity of element accumulation profiles for SLG and XCS was evaluated. Non-parametric Wilcoxon rank-sum tests were applied to compare concentrations of Cd, Pb, Hg, and Zn between paired species within each group, stratified by industrial and non-industrial sites. No statistically significant differences were identified (*p* > 0.05 for all elements and strata), suggesting comparable bioaccumulation behaviors. This similarity can be attributed to the shared mycorrhizal associations with coniferous hosts (for SLG) and broadleaf/coniferous hosts (for XCS), as well as analogous morphological and physiological traits that influence metal uptake. Aggregation was thus deemed appropriate to enhance analytical power while maintaining ecological relevance, without introducing bias into the species-specific interpretations.

## 3. Results

### 3.1. Sample Characteristics and Environmental Context

Table 2 presents the mean values, standard deviation (SD), as well as the minimum and maximum concentrations of the analyzed elements (Zn, Cd, Hg, Pb) in mushroom samples (SLG, LS, and XCS) broken down by sampling area: non-industrial and industrial, with a distinction between geographical direction and distance from the emission source. For element concentrations below LOQ, relevant information is provided in the table. In one SLG sample collected from an industrial area located 200–600 m west of the emission source, the Hg concentration was found to be above the LOQ, while in the other samples these values were below the LOQ. For these samples, the mean concentration and other parameters were not calculated.

The maximum permissible levels of Pb and Cd are regulated by Commission Regulation (EU) 2023/915 of 25 April 2023 on maximum levels for certain contaminants in food and repealing Regulation (EC) No 1881/2006 [50]. According to the Regulation, the maximum permissible Pb content in wild mushrooms is 0.80 mg·kg^−1^ f.m. (fresh mass), while the maximum permissible Cd content is 0.50 mg·kg^−1^ f.m. The maximum permissible levels of Hg compounds in mushrooms are stipulated in Commission Regulation (EU) 2018/73 of 16 January 2018, amending Annexes II and III to Regulation (EC) No 396/2005 of the European Parliament and of the Council with regard to maximum residue levels for mercury compounds in or on certain products [51]. The Regulation stipulates that the maximum permissible concentration of Hg in most wild mushroom species is 0.5 mg·kg^−1^ f.m., while for BE it is 0.9 mg·kg^−1^ f.m. The maximum permissible concentration of Zn is not regulated by law.

In a comprehensive analysis of 200 samples of mushrooms, it was found that 102 of these samples exceeded the maximum acceptable concentrations of the heavy metals: Cd, Pb, and Hg (Table S3—Supplementary Materials). Exceedances of the maximum permissible levels of the analyzed elements were recorded in mushrooms collected from all the locations studied, of which 33 (32%) were observed in mushroom samples collected from non-industrial areas, while 69 (68%) were found in samples from industrial areas. The lowest number of exceedances was recorded for Hg, and it was observed in a single sample of BE collected from a non-industrial area. It is noteworthy that the element with the highest concentrations in many of the mushroom species collected was Cd. In several cases, the concentrations of this element in mushroom samples taken from industrial areas exceeded the maximum permissible value by more than 1000%.

The concentrations of elements demonstrated variability across the groups. As illustrated in Table 3, the distribution of element concentrations is presented as the median (first quartile–third quartile). In the context of industrial sites, particularly those situated within a range of 200–600 m from emission sources, greater variability in Cd and Pb concentrations was observed, as evidenced by wider interquartile ranges.

### 3.2. Metals Concentrations in Mushroom Species Across Industrial and Non-Industrial Sites

The analysis encompassed three species of mushrooms, which were sampled in both industrial and non-industrial sites with a minimum of two specimens per group: SLG (15 non-industrial, 49 industrial), LS (3 non-industrial, 2 industrial), and XCS (10 non-industrial, 48 industrial). As detailed in Section 2.5, the aggregation of species within SLG and XCS is supported by statistical similarity in their accumulation profiles.

As shown in Table 4, industrial pollution does not consistently affect element concentrations across all mushroom species. For SLG and XCS, industrial sites significantly influenced most elements, with higher median Zn and Pb levels compared to non-industrial sites (SLG: Zn 8.52 vs. 6.85 mg·kg^−1^ f.m., *p* = 0.017; Pb 0.27 vs. 0.09 mg·kg^−1^ f.m., *p* < 0.001; XCS: Zn 22.79 vs. 14.30 mg·kg^−1^ f.m., *p* = 0.032; Pb 0.63 vs. 0.09 mg·kg^−1^ f.m., *p* < 0.001). Cd and Hg showed divergent patterns; SLG had lower Cd and Hg in industrial sites, while XCS exhibited higher Cd and no significant change in Hg.

In contrast, LS demonstrates no statistically significant differences in selected elements concentrations between industrial and non-industrial sites, with *p*-values ranging from 0.107 (Pb) to 0.800 (Zn). This absence of statistical significance can be attributed primarily to the limited sample size, which consists of three non-industrial and two industrial specimens. The reduced sample size severely limits the statistical power and consequently precludes the reliable detection of differences. For instance, Zn concentrations are comparable (8.93 mg·kg^−1^ f.m., IQR: 5.88–11.97 industrial vs. 8.70 mg·kg^−1^ f.m., IQR: 7.64–17.41 non-industrial; *p* = 0.800), and Pb shows a non-significant trend toward higher levels in industrial sites (0.22 mg·kg^−1^ f.m., IQR: 0.14–0.30 vs. 0.09 mg·kg^−1^ f.m., IQR: 0.09–0.09; *p* = 0.107).

### 3.3. Spatial Variation in Metals Concentrations Within Non-Industrial Sites

The analysis encompassed two species of mushrooms, sampled in non-industrial sites across Szydłowiec and Wadowice counties, with a minimum of two specimens per county: BE (33 Szydłowiec county, 5 Wadowice county) and *Boletus erythropus*—scarletina bolete (BER, 3 Szydłowiec county, 12 Wadowice county).

Spatial variation, as represented by Szydłowiec and Wadowice counties, does not consistently affect metals concentrations within non-industrial sites. For BE, the concentrations of Zn, Cd, and Pb remain comparable, indicating that county-specific factors have limited influence on this species. Conversely, *Boletus erythropus* demonstrates a substantial spatial variation for Zn (*p* = 0.018), exhibiting elevated concentrations in Szydłowiec county, though this difference is not statistically significant for other elements due to the limited sample size (n = 3) in Szydłowiec county (Table 5).

The inconsistent spatial effects indicate that other factors, such as species-specific elements uptake mechanisms, soil pH, organic matter content, or microsite variability, may play a more substantial role in determining element concentrations. For instance, the substantial Zn discrepancy in BER may be indicative of localized soil enrichment in Szydłowiec county or species-specific affinity for Zn uptake. Conversely, the absence of variations in BE for the majority of metals suggests a more uniform environmental baseline across counties. The limited sample size for BER in Szydłowiec county imposes constraints on the ability to discern variations, particularly for Cd and Pb, where trends (e.g., elevated Cd levels in Wadowice county) are evident.

### 3.4. Influence of Distance from Emission Sources on Elements Concentrations in Industrial Sites

As shown in Table 6, the influence of distance from emission sources on metal concentrations is species-specific and does not follow a consistent pattern of increase or decrease. In XCS, concentrations of Cd, Hg, and Pb were significantly higher at 200–600 m compared to 0–200 m (Cd: 4.64 vs. 2.48 mg·kg^−1^ f.m., *p* = 0.007; Hg: 0.048 vs. 0.037 mg·kg^−1^ f.m., *p* = 0.025; Pb: 0.93 vs. 0.41 mg·kg^−1^ f.m., *p* < 0.001). Zn concentrations were also higher at 200–600 m (27.67 vs. 22.35 mg·kg^−1^ f.m.) but the difference was not statistically significant (*p* = 0.436), likely due to high variability across sites. These results suggest that, for XCS, accumulation of certain elements tends to be greater at intermediate distances from emission sources.

In SLG samples, Zn concentrations showed a nearly significant increase at 200–600 m compared to 0–200 m (8.99 vs. 7.38 mg·kg^−1^ f.m.; *p* = 0.055), while Cd levels were significantly lower at the greater distance (0.13 vs. 0.19 mg·kg^−1^ f.m.; *p* = 0.015). Hg concentrations remained consistently low across both distance ranges (0.001 mg·kg^−1^ f.m.; *p* = 0.170), and Pb showed no significant difference (0.26–0.27 mg·kg^−1^ f.m.; *p* = 0.644), indicating minimal variation in uptake for these elements.

### 3.5. Influence of Geographical Position Relative to Emission Sources on Elements Concentrations in Industrial Sites

The analysis encompassed two species of mushrooms, sampled in industrial sites with a minimum of two specimens in both eastern and western geographical positions relative to emission sources. The following species were identified: SLG (26 east, 23 west) and XCS (23 east, 25 west).

As demonstrated in Table 7 a discernible correlation exists between the geographical location of mushroom sampling sites and the concentration of metals. This correlation is evident in the case of XCS. In XCS samples, higher concentrations of Zn (30.90 mg·kg^−1^ f.m. vs. 16.94 mg·kg^−1^ f.m.) and Cd (4.43 mg·kg^−1^ f.m. vs. 1.71 mg·kg^−1^ f.m.) were recorded in samples taken at locations east of the emission source (median differences of 13.96 mg·kg^−1^ f.m. and 2.72 mg·kg^−1^ f.m., respectively). Conversely, analogous relationships were not observed for Hg and Pb contamination of mushrooms.

No statistically significant correlations were found between certain element concentrations and the geographical location of SLG mushroom collection sites.

### 3.6. Multivariable Analysis of Metals Concentrations by Distance, Position, and Species in Industrial Sites

The robust linear regression (RLM) model evaluates the effects of distance from emission sources (0–200 m vs. 200–600 m), geographical position (east vs. west), mushroom species (SLG vs. XCS), and their two-way interactions on log-transformed concentrations of Zn, Cd, Hg, and Pb in industrial sites.

According to Table 8, the baseline Zn concentration for SLG at 0–200 m in the east position is 8.25 mg·kg^−1^ f.m. (β = 2.11, 95% CI: 1.87–2.35, *p* < 0.001). Distance to 200–600 m had a non-significant effect on SLG (β = 0.13, *p* = 0.369), while the west position slightly reduced concentrations (β = −0.20, *p* = 0.174). XCS showed a 4.44-fold higher baseline Zn level than SLG (β = 1.49, 95% CI: 1.11–1.86, *p* < 0.001). The interaction distance × species (200–600 m × XCS) was marginally significant (β = −0.41, *p* = 0.058), indicating a 0.66-fold decrease in Zn for XCS at 200–600 m relative to SLG. The interaction position × species (west × XCS) was significant (β = −0.47, *p* = 0.029), corresponding to a 0.63-fold decrease in Zn for XCS at west positions.

For Cd, the intercept (β = −1.68, 95% CI: −1.88–−1.48, *p* < 0.001) corresponds to 0.19 mg·kg^−1^ f.m. (e.g., e−1.68) for SLG at 0–200 m, east. Distance to 200–600 m reduces Cd concentration in SLG by 0.73-fold (β = −0.31, 95% CI: −0.55–−0.07, *p* = 0.014), while west position shows no effect (β = −0.17, 95% CI: −0.41–0.08, *p* = 0.179). XCS has a 20.72-fold higher baseline Cd level than SLG (β = 3.03, 95% CI: 2.71–3.35, *p* < 0.001). The interaction distance: 200–600 m × species: XCS increases Cd by 1.49-fold (β = 0.40, 95% CI: 0.05–0.76, *p* = 0.029), and position: west × species: XCS reduces it by 0.55-fold (β = −0.60, 95% CI: −0.96–−0.25, *p* = 0.001).

For Hg, the intercept (β = −6.91, 95% CI: −7.21–−6.60, *p* < 0.001) yields a baseline of approximately 0.001 mg·kg^−1^ f.m. (e−6.91) for SLG at 0–200 m, east. Distance to 200–600 m (β = 0.00, 95% CI: −0.36–0.36, *p* = 1.000) and west position (β = 0.00, 95% CI: −0.36–0.36, *p* = 1.000) show no effect for SLG. XCS has a 19.73-fold higher baseline Hg level than SLG (β = 2.98, 95% CI: 2.50–3.46, *p* < 0.001). The interaction distance: 200–600 m × species: XCS (β = 0.79, 95% CI: 0.25–1.32, *p* = 0.004) reveals a 2.20-fold increase (e.g., e0.79) in Hg for XCS at 200–600 m, and position: west × species: XCS (β = 0.50, 95% CI: −0.04–1.03, *p* = 0.068) indicates a non-significant 1.65-fold increase (e.g., e0.50).

For Pb, the intercept (β = −1.60, 95% CI: −1.87–−1.34, *p* < 0.001) corresponds to 0.20 mg·kg^−1^ f.m. (e.g., e−1.60) for SLG at 0–200 m, east. Distance to 200–600 m has no effect on SLG (β = −0.03, 95% CI: −0.35–0.29, *p* = 0.857), while west position slightly increases Pb concentration (β = 0.26, 95% CI: −0.06–0.59, *p* = 0.110). XCS shows a 1.99-fold higher baseline Pb level than SLG (β = 0.69, 95% CI: 0.27–1.12, *p* = 0.002). The interaction distance 200–600 m × species XCS increases Pb by 1.99-fold (β = 0.69, 95% CI: 0.21–1.16, *p* = 0.005), while the position west × species: XCS has no effect (β = −0.08, 95% CI: −0.55–0.40, *p* = 0.748).

XCS demonstrated significantly higher baseline concentrations of Zn (4.44-fold), Cd (20.72-fold), Hg (19.73-fold), and Pb (1.99-fold) than SLG, indicating superior elements accumulation capacity. In XCS samples collected at a distance of 200–600 from the emission source, an increase in Cd (1.49-fold) and Pb (1.99-fold) concentrations was observed, along with an non-significant decrease in Zn (0.66-fold) concentration. Furthermore, in XCS samples collected from locations west of the emission source, lower concentrations of both Zn (0.63-fold) and Cd (0.55-fold) were recorded. In SLG samples collected at locations between 200 and 600 m from the emission source, lower Cd concentrations (0.73-fold) are observed. The geographical location of the mushroom sampling points was found to be a significant factor in the concentration of metals in XCS, while no such relationship was identified for SLG.

### 3.7. Toxicological Risk Assessment

Table 9 and Table 10 present the detailed results of the estimated assessment of non-cancer health risks associated with dietary exposure of adults to selected metals. Zn, Cd, Pb, and Hg. The exposure assessment was based on two scenarios:

1. Scenario 1: the average daily consumption of edible wild forest mushrooms per capita—5.5 g per day [53];
2. Scenario 2: seasonal consumption of wild mushrooms among the population living in rural areas of large forest areas—96 g per day [54].

The two consumption scenarios were selected to reflect both average and high-end exposure conditions relevant to the Polish population. Scenario 1 (5.5 g/day) represents mean per capita consumption of wild mushrooms and is intended to estimate chronic background exposure. Scenario 2 (96 g/day) does not reflect habitual daily intake of the general population, but represents a conservative, seasonal exposure scenario applicable to rural and forest-adjacent populations, where intensive mushroom foraging and consumption during peak harvesting periods is a practice. This approach allows the identification of potential health risks among frequent consumers rather than population-wide averages. 

Three species of edible mushrooms were taken into account for this assessment: SLG, LS, and XCS, including the mean and maximum concentrations of Zn, Cd, Pb, and Hg.

The findings of Scenario 1 suggest that the hazard quotient (HQ) did not exceed 1 in any of the samples examined, indicating that chronic exposure to Zn, Cd, Pb, and Hg through the consumption of edible wild mushrooms does not pose a non-carcinogenic health risk to adults. In Scenario 2, where the estimated consumption of wild mushrooms is more than 17 times higher than in Scenario 1, it was demonstrated that the consumption of SLG mushrooms collected from industrial areas poses a non-carcinogenic health risk due to exposure to Hg (at a maximum concentration of 0.50 mg·kg^−1^ f.m.—HQ > 1). A health risk has also been demonstrated for the consumption of XCS mushrooms collected from both industrial and non-industrial areas. The ingestion of these mushrooms poses a non-carcinogenic health risk due to exposure to Cd. A notably elevated HQ ratio was documented for XCS consumption derived from industrial areas; for the mean Cd concentration of these samples (3.12 mg·kg^−1^ f.m.), HQ = 4.27817, while for the maximum Cd concentration (6.57 mg·kg^−1^ f.m.), HQ = 9.00754. The ingestion of XCS collected from non-industrial areas, where the maximum Cd concentration is 1.31 mg·kg^−1^ f.m., also poses a non-carcinogenic health risk (HQ = 1.79745).

Scenario 2 demonstrates that the consumption of both SLG and XCS mushrooms poses a non-carcinogenic health risk as a result of cumulative exposure to Zn, Cd, Pb, and Hg. For these species, the cumulative hazard index (HI) reached a value greater than 1 for the consumption of SLG mushrooms collected from industrial areas (for mean and maximum concentrations of the analyzed elements) and non-industrial areas (for maximum concentrations of the elements). Furthermore, the consumption of mushrooms of the XCS species from all analyzed areas also poses a non-carcinogenic health risk (for both mean and maximum concentrations of elements, the HI ranges between 1.212 and 11.012). Therefore, exceedances of HQ or HI values observed under Scenario 2 should be interpreted as indicative of potential health risks for frequent consumers during peak consumption periods rather than for the general population.

## 4. Discussion

In recent years, there has been a marked increase in demand for natural foods, including wild mushrooms, driven by consumer awareness of environmental and health issues [61,62,63]. Market analyses across Europe substantiate this trend. A nine-year observation in Spain demonstrated a statistically significant growth in both online searches and trade of wild mushrooms, including BE and *Lactarius deliciosus* [64]. In the Czech Republic, consumption increased from 2.4 kg per person in 2012 to 3.3 kg in 2021 [65]. This growing popularity can be attributed to the perception of mushrooms as natural and health-promoting foods. However, this assumption may be misleading, as mushrooms are efficient bioaccumulators of toxic metals, which can compromise consumer safety in polluted environments [66,67,68].

In this study, the concentrations of selected metals were compared in edible wild mushrooms collected from both industrial and non-industrial sites. The sampling locations were stratified according to their proximity to emission sources. This study utilized an analytical approach to investigate the prevalence of several frequently consumed species of mushrooms in Poland, namely *Suillus*, *Xerocomellus*, *Boletus*, and *Leccinum*. The analysis yielded a comprehensive spatial perspective on the transfer of pollutants, thereby extending and refining the methodological strategies conventionally employed in environmental and mycological assessments of mushrooms.

Nevertheless, the study is not without its limitations. The geographical scope of sampling was restricted to the southern part of Poland, which may limit geochemical representativeness. Moreover, the samples were obtained during a single growing season, which precludes temporal comparisons. In order to enhance the reliability of exposure assessments and provide a more comprehensive foundation for food safety recommendations, it is recommended that future multi-seasonal monitoring across a broader geographical gradient be conducted.

Finally, while the data were reported in mg·kg^−1^ f.m. in accordance with legal standards on maximum permissible values, comparison with other studies remains difficult, as most authors publish their findings in mg·kg^−1^ dry weight. It is important to note that the results were expressed in fresh mass (f.m.), which reflects the form in which mushrooms are consumed and enables a more accurate assessment of dietary exposure and public health risks. This perspective is rarely addressed in the literature.

The present study has demonstrated that environmental contamination by industrial elements does not exert a uniform effect on the concentration of these elements across all mushroom species. The aggregation of closely related species in SLG and XCS, confirmed by statistical analysis of similar accumulation profiles (as detailed in Section 2.5), allowed for robust comparisons while accounting for ecological similarities. The observed variability in the concentrations of Cd, Pb, Hg, and Zn indicates that the degree of element accumulation is predominantly determined by species-specific physiological mechanisms. Factors such as cell wall composition, hyphal structure, and metabolic pathways have been demonstrated to exert a significant influence on sorption and retention capacity. Furthermore, the physical and chemical properties of soils are of pivotal significance in determining metal bioavailability. For instance, Qin et al. [68] demonstrated that a one-unit change in soil pH can alter Cd accumulation in fruiting bodies by more than 40%.

Symbiotic relationships have also been demonstrated to play an essential role in shaping uptake efficiency. Bucurica et al. [67] reported that the transfer coefficient of Cd and Pb from soil to cap is on average 1.6 times higher in ectomycorrhizal fungi (e.g., BE, XCS) than in saprotrophic species such as *Agaricus bisporus* or *Armillaria mellea*. This phenomenon can be attributed to the enhanced absorptive surface area of ectomycorrhizal hyphae, as well as the presence of membrane transporters that are specifically adapted for this function.

The findings of this study provide direct evidence of interspecies variability under industrial pressure. Specifically, SL and SG, along with XCS, exhibited a marked tendency to accumulate significantly higher levels of Zn and Pb in industrial locations as compared to non-industrial ones. For *X. subtomentosus*, Cd concentrations were found to be up to seven times higher, while elevated Hg contents were observed in SL and SG from contaminated areas. These results not only confirm the differential bioaccumulation potential among species but also highlight the importance of species identity in assessing food safety risks.

The research conducted by Niezgoda et al. [38], involving the collection of edible wild mushrooms, was carried out in an industrial area that partially overlaps with the area where our own research was conducted—Tarnowskie Góry county, in the vicinity of the “Miasteczko Ślaskie” zinc smelter. In our own research within the area defined as industrial, Olkusz country was also identified. Niezgoda et al. demonstrated that the mean Cd content in the examined mushrooms was 0.98 mg·kg^−1^ f.m., which is nearly double the maximum permissible concentration. Moreover, several samples of BE exhibited concentrations of 3.35 and 3.01 mg·kg^−1^ f.m., respectively. In our own studies, the mean Cd concentration exhibited a dependence on the species of mushroom and the location of collection. The highest values were recorded in samples of XCS collected from an industrial area, where the average Cd concentration was 3.12 mg·kg^−1^ f.m., and the maximum concentration reached 6.57 mg·kg^−1^ f.m. In the study by Niezgoda et al., the average Pb content in the tested mushroom samples was 0.60 mg·kg^−1^ f.m., which is lower than the result obtained in our study—0.71 mg·kg^−1^ f.m. in XCS samples. In the study by Niezgoda et al., the concentration of Hg in mushrooms was on average 0.0274 mg·kg^−1^ f.m., while in the present study, the result was almost seven times higher—0.189 mg·kg^−1^ f.m. (SL and SG). Such a discrepancy in the results may be attributable to the different locations of the sampling sites, which were wider and more diverse in our study.

Research conducted by Stojek et al. [69] in northern Poland, in an area remote from anthropogenic emission sources, demonstrated that the bioaccumulation of metals is contingent on the species of mushroom and, to a lesser extent, on habitat conditions, including soil pH. The researchers presented the results of element and heavy metal concentrations in the dry weight of mushroom samples. It is not possible to directly compare these results with the results of our own research; however, it is possible to indicate existing relationships. The present study demonstrated that mushrooms belonging to the genus *Xerocomellus* exhibited a heightened propensity for accumulating heavy metals, specifically Pb and Cd, in comparison to other mushroom species. This finding was corroborated by the findings of our own research. The degree of contamination accumulation is influenced by both habitat characteristics and the concentration of the tested elements in the substrate.

For the study conducted by Bucurica et al. [67], samples of wild mushrooms representing the eight most commonly consumed species in Romania were collected. The mushrooms and samples of the substrate on which they grew were collected from industrialized and non-industrialized areas, indicating a similarity in the choice of location to our own research. The collection sites for the mushroom samples in Bucurica’s study encompassed two highly industrialized cities, three tourist destinations, and one transitional zone site. The researchers demonstrated not only species diversity in the degree of accumulation of the elements studied, but also a different interspecies tendency for their concentration in particular morphological parts of the fruiting body—the stem and cap. Notably, both soil and mushroom samples obtained from tourist areas believed to be free from anthropogenic pollution exhibited elevated concentrations of the studied elements, particularly Cd. When these concentrations were translated into an estimated health risk assessment, they indicated a potential threat to the health of consumers, particularly children. Our study has demonstrated that the consumption of both SL and SG, and XCS poses a non-carcinogenic health risk for adults as a result of cumulative exposure to Zn, Cd, Pb, and Hg.

Gałgowska and Pietrzak-Fiećko’s [70] study examined the concentrations of Cd and Pb in the dry mass of a selection of edible wild mushrooms harvested from the northern Polish region of Warmia and Mazury, a territory that has not been extensively industrialized. The presence of Cd and Pb was detected in all analyzed species of mushrooms, which is consistent with the results of our own research, in which Cd and Pb were found in most mushroom samples collected from non-industrialized areas. The heavy metal concentrations obtained by Gałgowska and Pietrzak-Fiećko did not exceed the levels permitted by European Union regulations; however, in the case of the porcini (BE), the values were relatively high.

A study by Demkova et al. [71] included an analysis of the Hg content in soil and the fruiting bodies of the mushroom *Amanita rubescens*. Samples were collected from various locations throughout the Slovak Republic. The highest concentrations of Hg were detected in soil and mushroom samples collected from industrial areas, which are post-mining areas. Our own studies also found statistically higher concentrations of Hg in mushroom samples collected from industrial areas (SL and SG). Demkova et al. demonstrated a correlation between elevated concentrations of Hg in the soil environment and its presence in the fruiting bodies of mushrooms. An analysis of Hg concentrations in different developmental stages of *A. rubescens* fruit bodies demonstrated that the concentration of this element increases with the age of the individual. Therefore, to ensure consumer safety, it is recommended to collect and consume younger fruit bodies.

This study makes a significant and original contribution to the fields of environmental toxicology, mycology and public health by addressing the safety of wild mushrooms in areas that have been industrially polluted. A pivotal innovation lies in the assessment of heavy metals and elements in fresh mass, which reflects the actual form in which mushrooms are consumed. The findings demonstrate that metal accumulation is strongly species-specific and influenced by habitat and spatial factors, thus challenging the common perception of wild mushrooms as inherently safe natural foods. From a public health perspective, these results support the provision of targeted recommendations for consumers and local authorities. These recommendations include guidance on species selection, harvest locations, and timing to minimize exposure to toxic elements. SL, SG, XCS were identified as species with the capacity to accumulate Zn, Cd, Pb, and Hg in areas influenced by industry.

Of particular significance is the direct relevance of the study for local populations who frequently harvest mushrooms from forested areas, thereby underscoring the necessity for increased consumer awareness regarding the potential risks associated with mushrooms from contaminated sites. Public education initiatives have the capacity to promote informed decision-making and safer harvesting practices, thereby ensuring that traditional foraging activities remain culturally and nutritionally beneficial while minimizing health hazards.

It is important to acknowledge the potential influence of seasonal and interannual variability in environmental factors, including rainfall, temperature, and mycelial growth stages, on the observed spatial patterns of selected element accumulation. Consequently, the risk assessment provided herein represents a snapshot based on the specific sampling periods and may not fully capture long-term exposure scenarios. It is recommended that future studies incorporating multi-year and multi-seasonal sampling be conducted to enhance our understanding of temporal fluctuations and to refine chronic exposure assessments. 

The present study establishes a practical and scientifically robust framework for assessing the health risks associated with the consumption of wild mushrooms. This framework integrates species-specific, environmental, and spatial factors, and it provides clear guidance for the development of evidence-based food safety policies, targeted market monitoring, and consumer education. The overarching aim of this guidance is to ensure effective protection of public health.

## 5. Conclusions

Exceedances of EU regulatory limits for Cd, Pb, and Hg were observed in multiple samples of mushrooms, including cases of co-occurrence of elevated concentrations of more than one element within individual fruiting bodies. The accumulation of elements manifested species-specific patterns, with *Xerocomellus chrysenteron* and *X. subtomentosus* displaying notably higher concentrations of Zn, Cd, Pb, and Hg compared to *Suillus luteus*, irrespective of their proximity or orientation with respect to pollution sources. Spatial factors, including distance from emission sources and prevailing wind direction, significantly influenced element concentrations in mushroom fruiting bodies, particularly in *X. chrysenteron* and *X. subtomentosus*. A risk assessment based on estimated intake scenarios indicated that consumption of *X. chrysenteron* and *X. subtomentosus* from both industrial and non-industrial areas may pose a non-carcinogenic health risk associated with Cd exposure (HQ > 1), with hazard index (HI) values exceeding safety thresholds in multiple cases. In view of the considerable species- and site-specific variability in element concentrations, it is recommended that future monitoring efforts incorporate ecological traits of mushroom species, spatial emission modelling, and soil–mushroom interactions within an integrated predictive framework.

## Figures and Tables

**Table 1:** Sampling characteristics for mushroom species across environmental conditions.

Characteristic	Site Sampling				
	Non-Industrial	Industrial			
		West Site, 0–200 m from Emission Source	East Site, 0–200 m from Emission Source	West Site, 200–600 m from Emission Source	East Site, 200–600 m from Emission Source
Mushroom Species (Acronym), n (%):					
AS	3 (3.0%)	0 (0%)	0 (0%)	0 (0%)	0 (0%)
BE	38 (38%)	0 (0%)	0 (0%)	0 (0%)	0 (0%)
BER	15 (15%)	0 (0%)	0 (0%)	0 (0%)	0 (0%)
CC	1 (1.0%)	0 (0%)	0 (0%)	0 (0%)	0 (0%)
LA	5 (5.0%)	0 (0%)	0 (0%)	0 (0%)	0 (0%)
LS	3 (3.0%)	0 (0%)	0 (0%)	2 (9.1%)	0 (0%)
MP	9 (9.0%)	0 (0%)	0 (0%)	1 (4.5%)	0 (0%)
RV	1 (1.0%)	0 (0%)	0 (0%)	0 (0%)	0 (0%)
SLG	15 (15%)	11 (38%)	13 (65%)	12 (55%)	13 (45%)
XCS	10 (10%)	18 (62%)	7 (35%)	7 (32%)	16 (55%)
Sampling County, n (%):					
Olkusz	0 (0%)	11 (38%)	13 (65%)	13 (59%)	13 (45%)
Szydłowiec	50 (50%)	0 (0%)	0 (0%)	0 (0%)	0 (0%)
Tarnowskie Góry	0 (0%)	18 (62%)	7 (35%)	9 (41%)	16 (55%)
Wadowice	50 (50%)	0 (0%)	0 (0%)	0 (0%)	0 (0%)

Note: AS = *Agaricus sylvaticus*, BE = *Boletus edulis*, BER = *Boletus erythropus*, CC = *Cantharellus cibarius*, LA = *Leccinum aurantiacum*, LS = *Leccinum scabrum*, MP = *Macrolepiota procera*, RV = *Russula virescens*, SLG = *Suillus luteus* and *Suillus grevillei*, XCS = *Xerocomellus chrysenteron* and *Xerocomellus subtomentosus*.

**Table 2:** Zn, Cd, Hg, and Pb concentrations [mg·kg^−1^ f.m.] in particular species of mushrooms.

	SLG				LS				XCS			
Non-Industrial	Mean	SD	Min	Max	Mean	SD	Min	Max	Mean	SD	Min	Max
Zn	7.10	2.93	4.32	15.67	11.25	5.36	7.64	17.41	15.00	5.69	8.49	25.34
Cd	0.28	0.15	0.06	0.68	0.16	0.09	0.06	0.24	0.58	0.42	0.09	1.31
Hg	0.019	0.010	0.002	0.035	0.011	0.007	0.003	0.016	0.049	0.050	0.002	0.129
Pb	0.19	0.08	0.13	0.24	<LOQ				0.33	0.28	0.10	0.64
Industrial												
Zn	9.63	7.60	4.35	57.66	8.93	4.31	5.88	11.97	28.53	19.06	4.58	77.40
Cd	0.17	0.08	0.01	0.52	0.36	0.06	0.31	0.40	3.12	1.79	0.17	6.57
Hg	0.189	0.272	0.001	0.500	<LOQ				0.051	0.031	0.011	0.199
Pb	0.34	0.28	0.11	1.88	0.22	0.11	0.14	0.30	0.71	0.39	0.04	1.95
West Site, 0–200 m from Emission Source												
Zn	6.35	1.58	4.35	9.28	NS				19.59	10.11	7.45	40.59
Cd	0.17	0.04	0.11	0.22					2.28	1.34	0.17	4.47
Hg	<LOQ								0.046	0.024	0.011	0.100
Pb	0.31	0.08	0.24	0.50					0.58	0.46	0.04	1.95
East Site, 0–200 m from Emission Source												
Zn	14.02	14.45	5.41	57.66	NS				29.96	23.62	4.58	54.96
Cd	0.17	0.15	0.03	0.52					2.11	2.14	0.65	5.86
Hg	0.283	0.308	0.065	0.500					0.084	0.057	0.035	0.199
Pb	0.50	0.54	0.11	1.88					1.00	0.18	0.89	1.39
West Site, 200–600 m from Emission Source												
Zn	9.14	2.17	6.20	13.16	8.93	4.31	5.88	11.97	46.24	25.92	13.00	77.40
Cd	0.17	0.06	0.01	0.22	0.36	0.06	0.31	0.40	3.08	0.93	1.77	3.99
Hg	-	-	-	-	<LOQ				0.033	0.007	0.020	0.040
Pb	0.28	0.10	0.13	0.42	0.22	0.11	0.14	0.30	0.49	0.26	0.28	1.03
East Site, 200–600 m from Emission Source												
Zn	8.83	1.60	6.67	12.00	NS				30.21	16.86	11.60	71.49
Cd	0.15	0.04	0.08	0.20					4.52	1.56	0.35	6.57
Hg	<LOQ								0.047	0.016	0.015	0.073
Pb	0.26	0.05	0.18	0.37					0.81	0.32	0.34	1.32

Note: NS—no samples, SD—standard deviation, SLG = *Suillus luteus* and *Suillus grevillea*, LS = *Leccinum scabrum*, XCS = *Xerocomellus chrysenteron* and *Xerocomellus subtomentosus*.

**Table 3:** Distribution of metals concentrations [mg·kg^−1^ f.m] by sampling characteristic according to the median (first quartile–third quartile).

Stratification Variable	Stratum	N	Zn	Cd	Hg	Pb
Sampling site characteristics	Industrial	100	11.24 (7.56–22.79)	0.35 (0.16–3.40)	0.001 (0.001–0.04)	0.33 (0.26–0.71)
	Non-industrial	100	12.04 (7.68–15.68)	0.35 (0.18–0.61)	0.13 (0.02–0.27)	0.09 (0.09–0.10)
County	Olkusz	50	8.49 (6.67–10.01)	0.16 (0.12–0.20)	0.001 (0.001–0.001)	0.27 (0.14–0.30)
	Szydłowiec	50	14.74 (12.41–17.83)	0.49 (0.31–0.68)	0.17 (0.07–0.29)	0.09 (0.09–0.18)
	Tarnowskie Góry	50	22.79 (14.22–41.05)	3.40 (1.42–4.47)	0.04 (0.03–0.06)	0.68 (0.40–0.97)
	Wadowice	50	8.32 (6.08–11.25)	0.22 (0.12–0.43)	0.05 (0.02–0.22)	0.09 (0.09–0.09)
Distance from emission source (m)	0–200	49	10.85 (7.38–22.35)	0.22 (0.18–2.48)	0.001 (0.001–0.04)	0.31 (0.26–0.47)
	200–600	51	11.84 (8.45–27.67)	0.40 (0.13–4.47)	0.02 (0.001–0.06)	0.43 (0.25–0.94)
Geographical position	East	49	11.60 (8.94–27.67)	0.22 (0.17–4.12)	0.001 (0.001–0.04)	0.31 (0.24–0.56)
	West	51	10.52 (6.41–22.35)	0.45 (0.15–1.71)	0.01 (0.001–0.06)	0.34 (0.26–0.89)

**Table 4:** Concentrations of selected metals [mg·kg^−1^ f.m.] in selected edible mushroom species.

Concentrations	N	n1	Non-Industrial	n2	Industrial	Median Difference (Industrial—Non-Industrial)	*p*	p_adj_	Effect Size (r)
			Median (IQR)		Median (IQR)				
SLG									
Zn	64	49	6.85 (4.75–7.42)	15	8.52 (6.68–10.01)	1.67 (95% CI: −0.62 to 3.96)	0.017	0.034	0.41
Cd	64	49	0.23 (0.19–0.34)	15	0.15 (0.12–0.19)	−0.08 (95% CI: −0.15 to −0.01)	<0.001	0.004	−0.67
Hg	64	49	0.019 (0.011–0.024)	15	0.001 (0.001–0.001)	−0.018 (95% CI: −0.022 to −0.014)	<0.001	0.004	−0.67
Pb	64	49	0.09 (0.09–0.09)	15	0.27 (0.18–0.30)	0.18 (95% CI: 0.10 to 0.26)	<0.001	0.004	0.67
LS									
Zn	5	2	8.70 (7.64–17.41)	3	8.93 (5.88–11.97)	Not applied ^1^	0.800	0.800	Not applied
Cd	5	2	0.19 (0.06–0.24)	3	0.36 (0.31–0.40)		0.200	0.240	
Hg	5	2	0.014 (0.003–0.016)	3	0.001 (0.001–0.001)		0.139	0.185	
Pb	5	2	0.09 (0.09–0.09)	3	0.22 (0.14–0.30)		0.107	0.161	
XCS									
Zn	58	10	14.30 (10.42–16.64)	48	22.79 (13.87–40.82)	8.49 (95% CI: 0.19 to 19.17)	0.032	0.055	0.43
Cd	58	10	0.47 (0.20–0.86)	48	3.53 (1.53–4.56)	3.06 (95% CI: 1.86 to 4.26)	<0.001	0.004	0.67
Hg	58	10	0.039 (0.004–0.084)	48	0.042 (0.032–0.061)	0.003 (95% CI: −0.060 to 0.066)	0.765	0.765	0.06
Pb	58	10	0.09 (0.09–0.10)	48	0.63 (0.40–0.95)	0.54 (95% CI: 0.34 to 0.73)	<0.001	0.004	0.67

^1^ Due to insufficient data. Note: SLG = *Suillus luteus* and *Suillus grevillea*, LS = *Leccinum scabrum*, XCS = *Xerocomellus chrysenteron* and *Xerocomellus subtomentosus*.

**Table 5:** Median (IQR) concentrations of selected metals [mg·kg^−1^ f.m.] in *Boletus edulis* and *Boletus erythropus* collected from non-industrial counties, with statistical comparisons.

Concentrations	N	n1	Szydłowiec County Median (IQR)	n2	Wadowice County Median (IQR)	Median Difference	*p*	p_adj_	Effect Size (r)
						(Wadowice—Szydłowiec)			
BE									
Zn	38	33	14.82 (12.41–17.71)	5	15.68 (14.88–16.21)	0.86 (95% CI: −0.73 to 2.45)	0.635	1.000	0.08
Cd	38	33	0.54 (0.42–0.73)	5	0.60 (0.58–0.64)	0.06 (95% CI: −0.05 to 0.17)	0.635	1.000	0.08
Hg	38	33	0.263 (0.113–0.370)	5	0.481 (0.332–0.538)	0.218 (95% CI: 0.034 to 0.402)	0.063	0.252	0.30
Pb	38	33	0.09 (0.09–0.10)	5	0.09 (0.09–0.10)	0.00 (95% CI: −0.01 to 0.01)	1.000	1.000	0.00
BER									
Zn	15	3	14.40 (14.35–25.36)	12	8.99 (6.62–11.61)	Not applied ^1^	0.018	0.144	Not applied ^1^
Cd	15	3	0.12 (0.07–0.16)	12	0.28 (0.11–0.42)	Not applied ^1^	0.233	0.622	Not applied ^1^
Hg	15	3	0.152 (0.021–0.168)	12	0.133 (0.018–0.236)	Not applied ^1^	0.945	1.000	Not applied ^1^
Pb	15	3	0.09 (0.09–1.07)	12	0.09 (0.09–0.10)	Not applied ^1^	0.643	1.000	Not applied ^1^

^1^ Due to insufficient data. Note: BE = *Boletus edulis*, BER = *Boletus erythropus*.

**Table 6:** Median (IQR) concentrations of metals [mg·kg^−1^ f.m.] in *Xerocomellus chrysenteron* and *Xerocomellus subtomentosus* and *Suillus luteus* and *Suillus grevillei*, stratified by distance from emission sources, with statistical comparisons.

Concentrations	N	n1	0–200 m Median (IQR)	n2	200–600 m Median (IQR)	Median Difference	*p*	p_adj_	Effect Size (r)
						(200–600 m–0–200 m)			
XCS									
Zn	48	25	22.35 (13.00–33.34)	23	27.67 (14.24–46.01)	5.32 (95% CI: −2.13 to 12.77)	0.436	0.498	0.15
Cd	48	25	2.48 (1.68–3.63)	23	4.64 (1.17–5.21)	2.16 (95% CI: 1.02 to 3.30)	0.007	0.028	0.38
Hg	48	25	0.037 (0.030–0.045)	23	0.048 (0.040–0.065)	0.011 (95% CI: 0.002 to 0.020)	0.025	0.050	0.30
Pb	48	25	0.41 (0.29–0.72)	23	0.93 (0.57–1.07)	0.52 (95% CI: 0.31 to 0.73)	<0.001	0.008	0.55
SLG									
Zn	49	24	7.38 (6.11–9.33)	25	8.99 (7.76–10.56)	1.61 (95% CI: −0.18 to 3.40)	0.055	0.088	0.27
Cd	49	24	0.19 (0.15–0.21)	25	0.13 (0.11–0.17)	−0.06 (95% CI: −0.09 to −0.03)	0.015	0.040	−0.35
Hg	49	24	0.001 (0.001–0.001)	25	0.001 (0.001–0.001)	0.00 (95% CI: 0.00 to 0.00)	0.170	0.227	0.00
Pb	49	24	0.27 (0.14–0.31)	25	0.26 (0.18–0.29)	−0.01 (95% CI: −0.08 to 0.06)	0.644	0.644	−0.07

Note: SLG = *Suillus luteus* and *Suillus grevillea*, XCS = *Xerocomellus chrysenteron* and *Xerocomellus subtomentosus*.

**Table 7:** Median (IQR) concentrations of elements [mg·kg^−1^ f.m.] in *Suillus luteus* and *Suillus grevillei* and *Xerocomellus chrysenteron* and *Xerocomellus subtomentosus*, stratified by geographical position relative to emission sources, with statistical comparisons.

Concentrations	N	n1	East Median (IQR)	n2	West Median (IQR)	Median Difference (West–East)	*p*	p_adj_	Effect Size (r)
SLG									
Zn	49	26	8.94 (7.38–10.80)	23	7.50 (5.89–9.48)	−1.44 (95% CI: −3.14 to 0.26)	0.083	0.166	−0.25
Cd	49	26	0.17 (0.13–0.19)	23	0.15 (0.11–0.20)	−0.02 (95% CI: −0.06 to 0.02)	0.388	0.517	−0.12
Hg	49	26	0.001 (0.001–0.001)	23	0.001 (0.001–0.001)	0.00 (95% CI: 0.00 to 0.00)	0.136	0.218	−0.20
Pb	49	26	0.25 (0.09–0.29)	23	0.27 (0.24–0.35)	0.02 (95% CI: −0.04 to 0.08)	0.075	0.200	0.26
XCS									
Zn	48	23	30.90 (16.42–49.42)	25	16.94 (10.52–31.21)	−13.96 (95% CI: −22.15 to −5.77)	0.024	0.096	−0.32
Cd	48	23	4.43 (3.50–5.05)	25	1.71 (1.11–3.55)	−2.72 (95% CI: −3.51 to −1.93)	<0.001	0.008	−0.52
Hg	48	23	0.040 (0.032–0.056)	25	0.045 (0.032–0.072)	0.005 (95% CI: −0.007 to 0.017)	0.337	0.539	0.14
Pb	48	23	0.57 (0.47–1.04)	25	0.72 (0.34–0.94)	0.15 (95% CI: −0.10 to 0.40)	0.728	0.728	0.07

Note: SLG = *Suillus luteus* and *Suillus grevillea*, XCS = *Xerocomellus chrysenteron* and *Xerocomellus subtomentosus*.

**Table 8:** Regression coefficients of the robust regression analysis of selected elements concentrations [mg·kg^−1^ f.m.] by distance, position, and species in industrial sites.

Predictor	Zn			Cd			Hg			Pb		
	β	95% CI	*p*	β	95% CI	*p*	β	95% CI	*p*	β	95% CI	*p*
Intercept [concentration (log) for SLG at 0–200 m, east position]	2.11	(1.87–2.35)	<0.001	−1.68	(−1.88–−1.48)	<0.001	−6.91	(−7.21, −6.60)	<0.001	−1.60	(−1.87–−1.34)	<0.001
Distance: 200–600 m [vs. Distance: 0 –200 m]	0.13	(−0.15–0.41)	0.369	−0.31	(−0.55–−0.07)	0.014	−1.20 × 10^−15^	(−0.36, 0.36)	1.000	−0.03	(−0.35–0.29)	0.857
Position: West [vs. Position: East]	−0.20	(−0.48–0.09)	0.174	−0.17	(−0.41–0.08)	0.179	−1.87 × 10^−16^	(-0.36, 0.36)	1.000	0.26	(−0.06–0.59)	0.110
Species: XCS [vs. Species: SLG]	1.49	(1.11–1.86)	<0.001	3.03	(2.71–3.35)	<0.001	2.98	(2.50, 3.46)	<0.001	0.69	(0.27–1.12)	0.002
Distance: 200–600 m × Species: XCS [vs. Distance: 0–200 m × Species: SLG]	−0.41	(−0.83–0.01)	0.058	0.40	(0.05–0.76)	0.029	0.79	(0.25, 1.32)	0.004	0.69	(0.21–1.16)	0.005
Position: West × Species: XCS [vs. Position: East × Species: SLG]	−0.47	(−0.89–−0.05)	0.029	−0.60	(−0.96–−0.25)	0.001	0.50	(−0.04, 1.03)	0.068	−0.08	(−0.55–0.40)	0.748

Note: SLG = *Suillus luteus* and *Suillus grevillea*, XCS = *Xerocomellus chrysenteron* and *Xerocomellus subtomentosus*.

**Table 9:** Average daily dose and health risks associated with multi- element exposure in adults from consumption of wild edible mushrooms (Scenario 1, intake rate—5.5 g/day).

Species	Site	Parameters of Concentration	Zn			Cd			Hg			Pb			HI
			C [mg·kg^−1^ f.m.]	ADD	HQ	C [mg·kg^−1^ f.m.]	ADD	HQ	C [mg·kg^−1^ f.m.]	ADD	HQ	C [mg·kg^−1^ f.m.]	ADD	HQ	
*SLG*	Industrial	Mean	9.63	0.00076	0.00252	0.17	0.00001	0.01300	0.19	0.00001	0.04941	0.19	0.00001	0.00412	0.069
		Max	57.66	0.00453	0.01510	0.52	0.00004	0.04070	0.50	0.00004	0.13095	1.88	0.00015	0.04112	0.228
	Non-industrial	Mean	7.10	0.00056	0.00186	0.28	0.00002	0.02187	0.02	0.000001	0.00496	0.19	0.00001	0.00404	0.033
		Max	15.67	0.00123	0.00410	0.68	0.00005	0.05343	0.04	0.000003	0.00927	0.24	0.00002	0.00524	0.072
*LS*	Industrial	Mean	8.93	0.00070	0.00234	0.36	0.00003	0.02789	<LOQ	-	-	0.22	0.00002	0.00480	0.035
		Max	11.97	0.00094	0.00314	0.40	0.00003	0.03143	<LOQ	-	-	0.30	0.00002	0.00655	0.041
	Non-industrial	Mean	11.25	0.00088	0.00295	0.16	0.00001	0.01278	0.01	0.000001	0.00290	<LOQ	-	-	0.019
		Max	17.41	0.00137	0.00456	0.24	0.00002	0.01855	0.02	0.000001	0.00412	<LOQ	-	-	0.027
*XCS*	Industrial	Mean	28.53	0.00224	0.00747	3.12	0.00025	0.24510	0.05	0.000004	0.01324	0.71	0.00006	0.01539	0.281
		Max	77.40	0.00608	0.02027	6.57	0.00052	0.51606	0.20	0.00002	0.05212	1.95	0.00015	0.04247	0.631
	Non-industrial	Mean	15.00	0.00118	0.00393	0.58	0.00005	0.04544	0.05	0.000004	0.01292	0.33	0.00003	0.00713	0.069
		Max	25.34	0.00199	0.00664	1.31	0.00010	0.10298	0.13	0.00001	0.03379	0.64	0.00005	0.01397	0.157

Note. Max—maximum concentration; C—concentration; ADD—Average Daily Dose; HQ—Hazard Quotient; HI—Hazard Index, SLG = *Suillus luteus* and *Suillus grevillea*, LS = *Leccinum scabrum*, XCS = *Xerocomellus chrysenteron* and *Xerocomellus subtomentosus*.

**Table 10:** Average daily dose and health risks associated with multi- element exposure in adults from consumption of wild edible mushrooms (Scenario 2, intake rate—96 g/day).

Species	Site	Parameters of Concentration	Zn			Cd			Hg			Pb			HI
			C [mg·kg^−1^ f.m.]	ADD	HQ	C [mg·kg^−1^ f.m.]	ADD	HQ	C [mg·kg^−1^ f.m.]	ADD	HQ	C [mg·kg^−1^ f.m.]	ADD	HQ	
*SLG*	Industrial	Mean	9.63	0.01320	0.04401	0.17	0.00023	0.22687	0.19	0.00026	0.86248	0.19	0.00026	0.07187	1.205
		Max	57.66	0.07908	0.26359	0.52	0.00071	0.71040	0.50	0.00069	2.28571	1.88	0.00258	0.71771	3.977
	Non-industrial	Mean	7.10	0.00974	0.03246	0.28	0.00038	0.38181	0.02	0.00003	0.08655	0.19	0.00025	0.07048	0.571
		Max	15.67	0.02149	0.07163	0.68	0.00093	0.93257	0.04	0.00005	0.16174	0.24	0.00033	0.09143	1.257
*LS*	Industrial	Mean	8.93	0.01224	0.04080	0.36	0.00049	0.48686	<LOQ	-	-	0.22	0.00030	0.08381	0.611
		Max	11.97	0.01642	0.05472	0.40	0.00055	0.54857	<LOQ	-	-	0.30	0.00041	0.11429	0.718
	Non-industrial	Mean	11.25	0.01543	0.05143	0.16	0.00022	0.22310	0.01	0.00002	0.05069	<LOQ	-	-	0.325
		Max	17.41	0.02388	0.07961	0.24	0.00032	0.32384	0.02	0.00002	0.07197	<LOQ	-	-	0.475
*XCS*	Industrial	Mean	28.53	0.03912	0.13041	3.12	0.00428	4.27817	0.05	0.00007	0.23116	0.71	0.00097	0.26860	4.908
		Max	77.40	0.10615	0.35383	6.57	0.00901	9.00754	0.20	0.00027	0.90971	1.95	0.00267	0.74133	11.012
	Non-industrial	Mean	15.00	0.02057	0.06855	0.58	0.00079	0.79308	0.05	0.00007	0.22544	0.33	0.00045	0.12444	1.212
		Max	25.34	0.03475	0.11584	1.31	0.00180	1.79745	0.13	0.00018	0.58971	0.64	0.00088	0.24381	2.747

Note. Max—maximum concentration; C—concentration; ADD—Average Daily Dose; HQ—Hazard Quotient; HI—Hazard Index, SLG = *Suillus luteus* and *Suillus grevillea*, LS = *Leccinum scabrum*, XCS = *Xerocomellus chrysenteron* and *Xerocomellus subtomentosus*.

## Data Availability

The datasets generated during the current study are available from the corresponding author on reasonable request.

## Supplementary Materials

The following supporting information can be downloaded at: https://www.mdpi.com/article/10.3390/toxics14010036/s1, Table S1: Geographic location of the sampling areas; Table S2: Method description and measuring conditions of the spectrometers ICP-OES, ET-AAS, and CV-AFS; Table S3: Mushroom samples and species with Cd, Pb or Hg concentrations exceeding the maximum permissible levels.
