# Supplementary material for: Spatial Variation in Cd, Pb, Hg, and Zn Accumulation in Edible Wild-Growing Mushroom Species from Different Environmentally Loaded Areas in Southern Poland: Risk Assessment and Implications for Consumer Safety
Source: Toxics. 2025 Dec 29;14(1):36. doi: 10.3390/toxics14010036 (PMC12845654; doi:10.3390/toxics14010036)
Supplement: Supplementary file 1 [file toxics-14-00036-s001.zip › toxics-4029533-supplementary.pdf]

Table S1. Geographic location of the sampling areas.

Site Sampling	Latitude (°N)	Longitude (°E)
Tarnowskie Góry county	50.4976-50.5067	18.9098-18.9442
Olkusz county	50.2728-50.2889	19.4575-19.5184
Wadowice county	49.7957-49.8017	19.4164-19.4536
Szydłowiec county	51.1912-51.2015	20.7900-20.8322

Table S2. Method description and measuring conditions of the spectrometers ICP-OES, ET-AAS, and CV-AFS.

Element	**Zn**	**Cd**	**Pb**	**Hg**
Analytical methods	ICP-OES	ET-AAS		CV-AFS
Line [nm]	213.857	228.8	217.0	254.0
**Calibration curve**				
Calibration range [mg/L]; [μg/L]*	0.50 - 3.00	5.00 - 20.00	10.00 - 40.00	0.00 – 20.00
Calibration of failure criteria : R^2	≥ 0.995	≥ 0.995		≥ 0.995
RSD for standards [%]	< 5%	< 10 %		< 10 %
Calibration type	maximum	least squares		least squares
**Characteristics of the method**				
R^2 value	0.999	0.998	0.998	0.999
Recovery [%]	87 - 95	83 - 118	80 - 116	93 - 115
RSD [%]	4.6	< 20 %		< 10 %
LOD [$mg \cdot kg^{-1}$]	0.42	0.005	0.053	0.0005
LOQ [$mg \cdot kg^{-1}$]	0.69	0.01	0.10	0.001
**Measuring conditions of the spectrometer**				
Slit width [nm]	-	0.50	1.00	-
Lamp current [mA]	-	3.00	5.00	-
Background correction	-	deuterium		-
Sample volume [μL]	-	20.00	20.00***	-
Gas flow (Ar 99,999%) [L/min]	12-13	3		2.50
Radio frequency power [W]	1000	-		-
Type of nebulizer	teflon	-		-
Peristaltic pump speed [rpm]**	15	-		100
Spectrometer ambient temperature [°C]	18-24 ± 2	10-35		5-40
Air humidity [%]	20-80	3-30		< 80 up to 31°C

* [mg/L] for ICP-OES; [μg/L] for ET-AAS and CV-AFS

** % for CV-AFS

*** 16.00 + 4.00 μL matrix modifier - $NH_4H_2PO_4$ & 600μg/mL $Mg(NO_3)_2$ in 2% HNO_3

Table S3. Mushroom samples and species with Cd, Pb or Hg concentrations exceeding the maximum permissible levels.

Characteristic	Number of Samples	Mushroom Species	Concentration Range [mg·kg^{-1} f.m.]	% of Maximum Permissible Level
**Non-industrial**				
Cd	32	BE (n=24)	0.52-1.43	104-286
		XCS (n=4)	0.81-1.31	162-262
		BER (n=1)	1.22	244
		MP (n=1)	0.97	194
		LA (n=1)	0.55	110
		SLG (n=1)	0.68	136
Pb	-	-	-	-
Hg	1	BE (n=1)	0.914	102
**Industrial**				
West Site, 0-200 m from Emission Source				
Cd	16	XCS (n=16)	0.75-4.47	150-894
Pb	4	XCS (n=4)	0.86-1.95	108-244
Hg	-	-	-	-
East Site, 0-200 m from Emission Source				
Cd	7	XCS (n=7)	1.77-3.99	354-798
Pb	1	XCS (n=1)	1.03	129
Hg	-	-	-	-
West Site, 200-600 m from Emission Source				
Cd	9	XCS (n=7)	0.65-5.86	130-1172
		SLG (n=1)	0.52	104
		MP (n=1)	1.38	276
Pb	10	XCS (n=7)	0.89-1.39	111-174
		SLG (n=2)	0.97-1.88	121-235
		MP (n=1)	1.16	145
Hg	-	-	-	-
East Site, 200-600 m from Emission Source				
Cd	15	XCS (n=15)	1.47-6.57	294-1314
Pb	7	XCS (n=7)	0.93-1.32	116-165
Hg	-	-	-	-

Note: BE = *Boletus edulis* L. BER = *Boletus erythropus*, LA = *Leccinum aurantiacum*, MP = *Macrolepiota procera*, SLG = *Suillus luteus* & *Suillus grevillea*, XCS = *Xerocomellus chrysenteron* & *Xerocomellus subtomentosus*
